# IL-17 promoted the inhibition of medulloblastoma in mice by splenocyte injection

**DOI:** 10.1186/s40001-015-0191-8

**Published:** 2015-12-18

**Authors:** Ping Zhou, Qilin Zhang, Yao Zhao, Hongying Sha, Xiaoyun Cao, Yongfei Wang

**Affiliations:** Department of Neurosurgery, Huashan Hospital, Fudan University, Shanghai, 200040 China; Institute of Biomedical Sciences, Fudan University, Shanghai, 200032 China

**Keywords:** Th17 cells, IL-17, Inflammatory factors, Medulloblastoma, Anti-tumor immunity

## Abstract

**Background:**

Interleukin 17 (IL-17) is a proinflammatory cytokine produced by a new subset of activated CD4+ T cells, Th17 cells. We previously showed that increased Th17 cell populations were presented in human medulloblastoma-infiltrating T cells and peripheral blood. In this study, we attempted to address the possible role of Th17 cells in the biologic activity of IL-17 for tumor control.

**Methods:**

We grafted fresh surgically obtained medulloblastoma into syngeneic athymic nude/nude mice. We intrapertonially injected splenocyte and murine IL-17 in mice on the second day. The tumor volume and the life spans of the mice were measured. Meanwhile, the IL-17, IL-6, IL-23, Ccl2, Ccl20 and IFN-gamma expression in the tumors was also examined by real-time PCR, Western blot and enzyme-linked immunosorbent assay.

**Results:**

We found that medulloblastoma growth in IL-17-injected mice was significantly inhibited compared to the non-IL-17 treated mice. In contrast to the IL-17 antitumor activity observed in mice injected with splenocytes, we observed that IFN-gamma, IL-6, IL-23, Ccl2, and Ccl20 proteins were significantly increased in tumor tissues of mice injected with IL-17.

**Conclusions:**

These experiments suggest that IL-17 may promote splenocyte antitumor activity in medulloblastoma. We postulate that IL-17’s antitumor activity may be related to the increased protein levels of IFN-gamma, IL-6, IL-23, Ccl2, and Ccl20.

## Background

Medulloblastoma is a malignant tumor of the cerebellum and one of the most frequent malignant tumors in children. Medulloblastoma is usually associated with a worse prognosis than other pediatric tumors. Moreover, it is the most common malignant pediatric brain tumor. The median age of children at diagnosis is 5-year old, and the tumor age range extends into young adulthood. Current therapies have serious adverse effects such as postoperative mutism, neurocognitive deficits, endocrinopathies, and sterility [1–7]. There is compelling evidence showing the immune system’s vital role regarding diverse malignant tumors, as it will affect cancer cell proliferation, migration, and survival [4, 8, 9].

Th17 cells are a new member of the CD4+ effector T cell family and characterized as preferential producers of IL-17A, IL-17F, IL-21, and IL-22. Retinoid orphan nuclear receptor (RORC), which encodes the ortholog RORyt, is a key regulator in Th17 cell differentiation. IL-23 and IL-6 also play a role in human Th17 cells in vitro. Th17 cell differentiation is also less well understood in vivo and in vitro. Th17 cells play a physiological role in promoting host defense against infectious agents and sometimes contribute to autoimmune disease [10, 11]. Recently, accumulated evidence showed that Th17 cells and interleukin-17 have increased frequencies within several tumor types, such as medulloblastoma and ovarian cancer. However, the role of Th17 and IL-17 in tumor development has been controversial. Th17 and IL-17 have beneficial, detrimental, direct, and indirect effects on some tumors’ development. Th17’s role in tumors is dependent on the tumor’s classification and microenvironment. Many tumor cell types also bear IL-17 receptor alpha (IL-17RA), the specific receptor for IL-17 [12–15].

The accumulated evidence demonstrated that IL-17 may have a direct impact on the tumor cells’ biological behavior in the local microenvironment. Some settings demonstrated that IL-17 and Th17 had an inverse correlation with certain cancer’s progression in clinical studies. Much evidence showed that IL-17 increased vascular endothelium and tumor neoangiogenesis and promoted tumor cell development in mice [14, 15]. However, other settings demonstrated that IL-17 induced tumor suppression and even eradication by facilitating the immune cell recruitment in vivo [16, 17]. Our previous studies have shown increased IL-17+ T cell numbers within medulloblastoma tumors [18]. However, the direct effects and underlying mechanisms of IL-17 in medulloblastoma cell growth remains elusive. Thus, in this study, we attempted to do a tentative exploration in the role of IL-17 on medulloblastoma in nude mice with splenocyte injection. We also investigated the expressions of IL-17-related cytokines and the survival rates of mice bearing medulloblastoma.

## Methods

### Mice

We obtained 6- to 8-week-old female wild-type Balb/c mice and Balb/c athymic nude/nude mice from the Laboratory Animal Center of Fudan University. Mice were maintained under pathogen-free conditions in this study. This study was approved by the Huashan Institutional Review Board, Fudan University, Shanghai, China.

### Patients and tumor cells

According to the previous study [19], we collected tumor tissues during operation. No patient received radiotherapy or chemotherapy preoperatively. This study was approved by the Ethical Committee of Fudan University, and we obtained written informed consent from all individuals involved in this research.

We cultured tumor cells in high-glucose Dulbecco’s modified Eagle medium (DMEM) supplemented with 10 % heat-inactivated fetal bovine serum (FBS), 2 mM l-glutamine, 100 units/mL penicillin, 100 μg/mL streptomycin, and 0.01 % mercaptoethanol. All cells were maintained at 37 °C in a humidified incubator in 5 % CO_2_. The cells were then collected and resuspended at 1 × 10^6^/100 μL in PBS, and we then injected a 100-μL suspension into mice shoulders.

### Splenocyte preparation and proliferation

According to the previous study [20], splenocytes (2 × 10^7^/mL) were purified and stimulated with 2 μg/mL ConA in complete DMEM [DMEM with 10 % fetal calf serum (FCS), 10 μM HEPES, 50 μM β-mercaptoethanol, 2 mM l-glutamine, and 50 IU/mL penicillin–streptomycin] in 25-mL flasks for 72 h. Splenocytes were then washed three times with PBS (pH 7.4). Freshly isolated splenocytes (2 × 10^7^ per well) were cultured in a flat-bottomed plate in triplicate in complete DMEM with ConA (2 μg/mL) at 37 °C in 5 % CO_2_ for 72 h.

### Mice grouping

Thirty animals were randomly divided into the saline-injected group (*n* = 10), splenocyte-injected group (*n* = 10), and splenocyte-IL-17-injected group. Medulloblastoma challenge time was used as the baseline (time point 0). In the splenocytes-injected group, the mice were intraperitoneally injected with 5 × 10^7^ allogenic splenocytes in 2 mL sodium butyrate solution 48 h after medulloblastoma challenge. While in the splenocyte-IL-17-injected group, 5 × 10^7^ allogenic splenocytes and IL-17 (ProSpec) (80 ng/mL) in 2 mL sodium butyrate solution were intraperitoneally injected at the same time. The injected doses were recommended by other authors and were approved by our pilot experiment [20, 21]. The mice in the saline-injected group were injected intraperitoneally with the same volume of normal saline.

Then, four mice in each group were randomly euthanized for tumor sampling  at 28 days. Tumors from the euthanized mice in each group were used to examine IL-6, IL-23, Ccl2, and Ccl20 protein expressions by Western blotting and real-time PCR. The remaining six mice per group were observed for survival duration.

### Tumor growth inhibition assay in mice

After four mice in each group were randomly euthanized, we observed the remaining six mice per group for survival duration after medulloblastoma challenge. We measured tumor diameter (in millimeters) twice every week and determined tumor volume according to the following formula: volume = (*a* × *b*)^2^/2 (*a* long diameter; *b* short diameter).

### Survival analysis

The remaining six mice in each group were used to measure life span. We analyzed survival curves using the non-parametric Kaplan–Meier method.

### Western blotting

To determine the expressions of inflammatory factors and cytokines, we analyzed the fresh tissue samples of these medulloblastoma xenografts by Western blotting. Cell lysates were isolated in a protein extraction buffer containing 150 mM NaCl, 20 mM Tris (pH 7.5), 5 mM EDTA, 0.1 % Triton X-100, 5 % glycerol, and 2 % SDS. After incubating at 4 °C for 30 min, the protein concentrations were centrifuged at 12,000 rpm for 30 min. Protein concentrations were determined using Bradford assay. Proteins were then denatured in a sample buffer containing 2-mercaptoethanol and bromophenol blue for 10 min at 95 °C. Equal protein amounts were resolved by SDS-PAGE and then transferred to PVDF membranes. The membranes were blocked in 5 % skimmed milk in tris-buffered saline containing 0.1 % Tween 20 (TBST) and were then incubated overnight at 4 °C with the primary antibodies (Santa Cruz Biotechnology, Inc). After being washed with PBS three times, the membranes were incubated in secondary antibody at room temperature. We detected protein band intensity using an enhanced chemiluminescence detection system.

### Real-time RT-PCR

We dissected medulloblastoma xenograft samples and extracted mRNA using UNIzol reagent treated with RNase-free DNase I (Takara, Japan). Reverse transcription was performed with Omniscript reverse transcriptase (QIAGEN). The 20-μL reactions contained: DNase-treated RNA, 1 μM random hexamer primer, deoxynucleoside triphosphate mix, 1 U of RNase inhibitor (Ambion), and Omniscript reverse transcriptase. Incubation conditions were those suggested by the manufacturer: 37 °C for 1 h and 93 °C for 5 min. We performed real-time PCR analysis with SYBR Green I (Takara, Japan). The 50-μL reactions contained: cDNA; 0.5 μM of primers specific for primer sequences: Il-6 forward, 5′-TGATGGATGCTACCAAACTGG-3′ and reverse, 5′-TTGGTCCTTAGCCACTCCTTC-3′ (2.5 mM MgCl_2_); Il23a forward, 5′-AATGCTATGGCTGTTGCCCTG-3′ and reverse, 5′-CGGATCCTTTGCAAGCAGAAC-3′(3.5 mM MgCl_2_); Ccl2 forward, 5′-TGATCCCAATGAGTAGGCTGG-3′ and reverse, 5′-TGCTTGAGGTGGTTGTGGAA-3′ (2.5 mM MgCl_2_); Ccl20 forward, 5′-TCTTCCTTGCTTTGGCATGG-3′ and reverse, 5′-TCTGCTTTGGATCAGCGCA-3′ (2.5 mM MgCl_2_); GAPDH forward, 5′-ACCACAGTCCATGCCATCAC-3′ and reverse, 5′- TCCACCACCCTGTTGCTGTA-3′ (2.5 mM MgCl_2_). PCR was performed in ABI PRISM 7900HI (Applied Biosystems), and cycle conditions were 50 °C for 2 min and 95 °C for 10 min, followed by 40 cycles of 95 °C for 15 s and 60 °C for 1 min. Endogenous control (GAPDH) was used for each sample in the same plate. Gene expression was quantified by the 2^−ΔΔCt^ method.

### Enzyme-linked immunosorbent assay (ELISA)

Sera and ascitic fluid were collected from three groups of mice on day 14. The serum and ascitic fluid samples were tested for the productions of IL-17 and IFN-gamma using ELISA kits, according to the manufacturer’s instructions (Quantikine).

### Statistical analysis

We performed statistical analysis using SPSS 16.0 for Windows software. The reported values were expressed as means ± standard deviations. Statistical analysis was performed using Student’s *t* test and *χ*^2^ test, and we analyzed the survival curves using the non-parametric Kaplan–Meier method. Differences were judged as significant at a value of *P* < 0.05.

## Results

### IL-17 enhanced splenocyte injections’ inhibition on medulloblastoma growth in vivo

As shown in our previous work, the Th17 population was increased significantly both in peripheral blood and tumor-infiltrating T cells of medulloblastoma patients. To investigate the role of Th17 in medulloblastoma development in vivo, we challenged Balb/c nu/nu mice with medulloblastoma injected into the right shoulder subcutaneously. Then, we injected the mice with allogenic splenocytes intraperitoneally 2 days after medulloblastoma challenge. We added IL-17 (80 ng/mL) to the splenocyte suspension after injection in the test group but not in the control group.

**Fig. 1:**
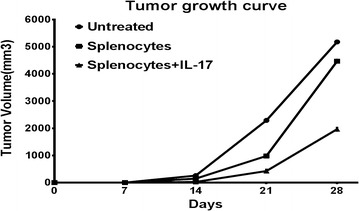
Tumor growth curve. The medulloblastoma tumor growth rate was decreased in the mice injected with splenocytes with IL-17. The mean tumor volume on day 28 in the splenocyte-injected group with IL-17 was smaller than in the splenocyte-injected group without IL-17 (1966.81 ± 378.16 mm^3^ in IL-17-treated group vs 4468.18 ± 923.64 mm^3^ in non-IL-17-treated group; *P* < 0.01). Meanwhile, the mean tumor volume in non-splenocyte-injected mice was the largest (6012.45 ± 572.95 mm^3^)

On day 28 after medulloblastoma challenge, mice injected with IL-17 exhibited significantly smaller tumor sizes compared to the non-IL-17 treated mice (1966.81 ± 378.16 mm^3^ in IL-17-treated group vs 4468.18 ± 923.64 mm^3^ in the non-IL-17-treated group; *P* < 0.01). The tumor growth curves illustrated that the medulloblastoma cells grew slowly in the IL-17-treated group on days 14, 21, and 28 after medulloblastoma challenge (Fig. 1).

Meanwhile, on day 28 after the challenge, the mean medulloblastoma volumes in the Balb/c nu/nu mice without splenocyte injection exhibited bigger tumor sizes compared to the mice in both groups with splenocyte injection. The medulloblastoma growth curves showed that the mean tumor size in the splenocyte-injected mice was bigger than the mice (with or without IL-17) injected with splenocytes on days 14, 21, and 28 after tumor challenge, as shown in Fig. 1. However, the difference was significant between the non-splenocyte-injected group and the splenocyte-injected group without IL-17 (6012.45 ± 572.95 vs 4468.18 ± 923.64 mm^3^; *P* < 0.01, Fig. 1).

To investigate the effect of IL-17 alone in tumor growth in Balb/c nu/nu mice, we injected the same dose of IL-17 (80 ng/mL) in Balb/c nu/nu mice without splenocyte injection. There was no tumor size difference in the Balb/c nu/nu mice without splenocyte injection between the IL-17-treated group and non-IL-17-treated group on days 14, 21, and 28 (data not shown). Therefore, IL-17’s effect on medulloblastoma inhibition is splenocyte dependent in Balb/c nu/nu mice. This suggested that IL-17 may enhance the splenocyte injections’ inhibition of medulloblastoma growth in vivo in nude mice, and IL-17 alone may have no significant effect on medulloblastoma.

### Splenocyte injection prolonged nude mice life spans

**Fig. 2:**
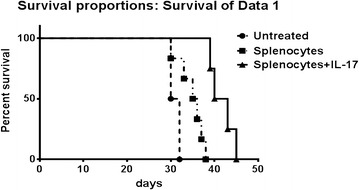
IL-17 increased survival time of mice injected with splenocytes. The splenocyte-injected group lived longer than those in the non-splenocyte-injected group (*P* < 0.05). Moreover, in splenocyte-injected mice, the IL-17-treated group had a significantly increased survival time compared to the non-IL-17-treated group (*P* < 0.01)

The remaining six mice in each group were used to measure life span. Survival curves were analyzed using the non-parametric Kaplan–Meier method. As shown in Fig. 2, the tumor survival curves illustrated that the splenocyte-injected group lived longer than those in the non-splenocyte-injected group (*P* < 0.05). Additionally, between the two splenocyte-injected groups, the IL-17-treated group had an increased survival time compared to the non-IL-17-treated group, and the difference was significant in statistics by the Kaplan–Meier method (*P* < 0.01). It suggested that IL-17 may enhance the therapeutic effects of splenocyte injection against medulloblastoma in nude mice.

### IL-6 and IL-23 were overexpressed in IL-17-treated mice

**Fig. 3:**
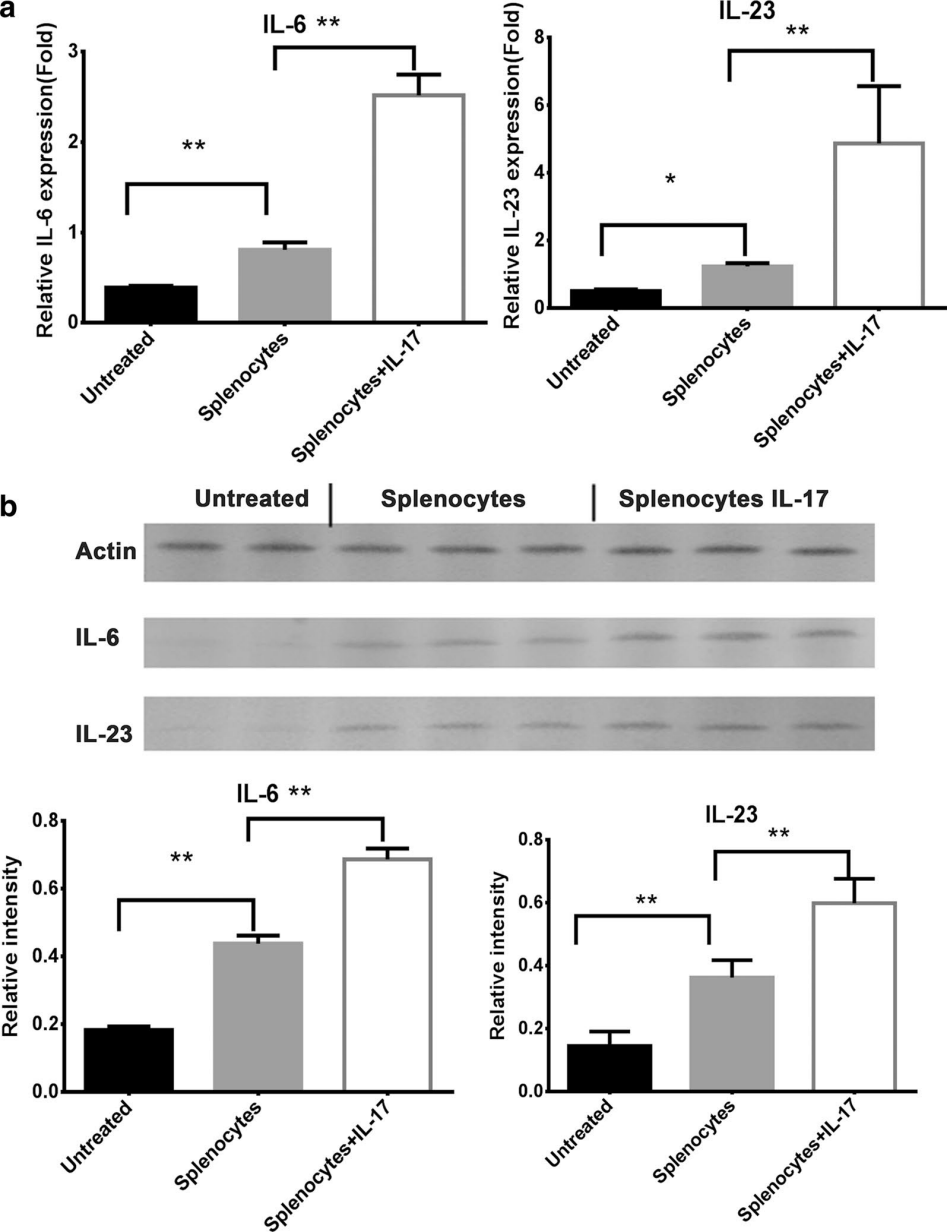
**a** The medulloblastoma xenografts were removed from euthanized mice of all groups on day 28, and cell lysates were isolated for protein and mRNA extraction. Total RNA was extracted, and the mRNA levels of IL-6 and IL-23 were analyzed using real-time PCR. Endogenously expressed GAPDH amplification in the same samples was used for a loading control. The experiment was performed three times with consistent results. *p* values were calculated with the *t* test; *p < 0.05; **p < 0.01. **b** Cell lysates were isolated in a protein extraction, and Ccl2 and Ccl20 protein was analyzed using Western blot. The similar expressions of Ccl2 and Ccl20 proteins in medulloblastoma cells were confirmed by Western blot assay and densitometry assessment. *p* values were calculated with the *t* test; *p < 0.05; **p < 0.01

Because IL-6 and IL-23 are cytokines closely related to IL-17, we also examined the expression levels of IL-6 and IL-23 in the medulloblastoma xenografts by real-time PCR and Western blot to investigate the microenvironment changes induced by IL-17. The medulloblastoma xenografts were removed from euthanized mice of all groups on day 28, and cell lysates were isolated for protein and mRNA extraction. As shown in Fig. 3, the Western blot results were in accordance with that of real-time PCR. Compared to the non-splenocyte-injected group, results obtained from splenocyte-injected mice showed that IL-6 and IL-23 were overexpressed in tumors. Moreover, the results also revealed that the in the splenocyte-injected group, the IL-6 and IL-23 expression levels were significantly higher in the IL-17-treated group than the non-IL-17-treated group. These results implicated that splenocyte injection may promote the expressions of IL-6 and IL-23 in medulloblastoma xenografts in nude mice, and IL-17 may enhance the effects of splenocyte injection.

### IL-17 enhanced the antitumor effect of splenocyte injection on Ccl2 and Ccl20

**Fig. 4:**
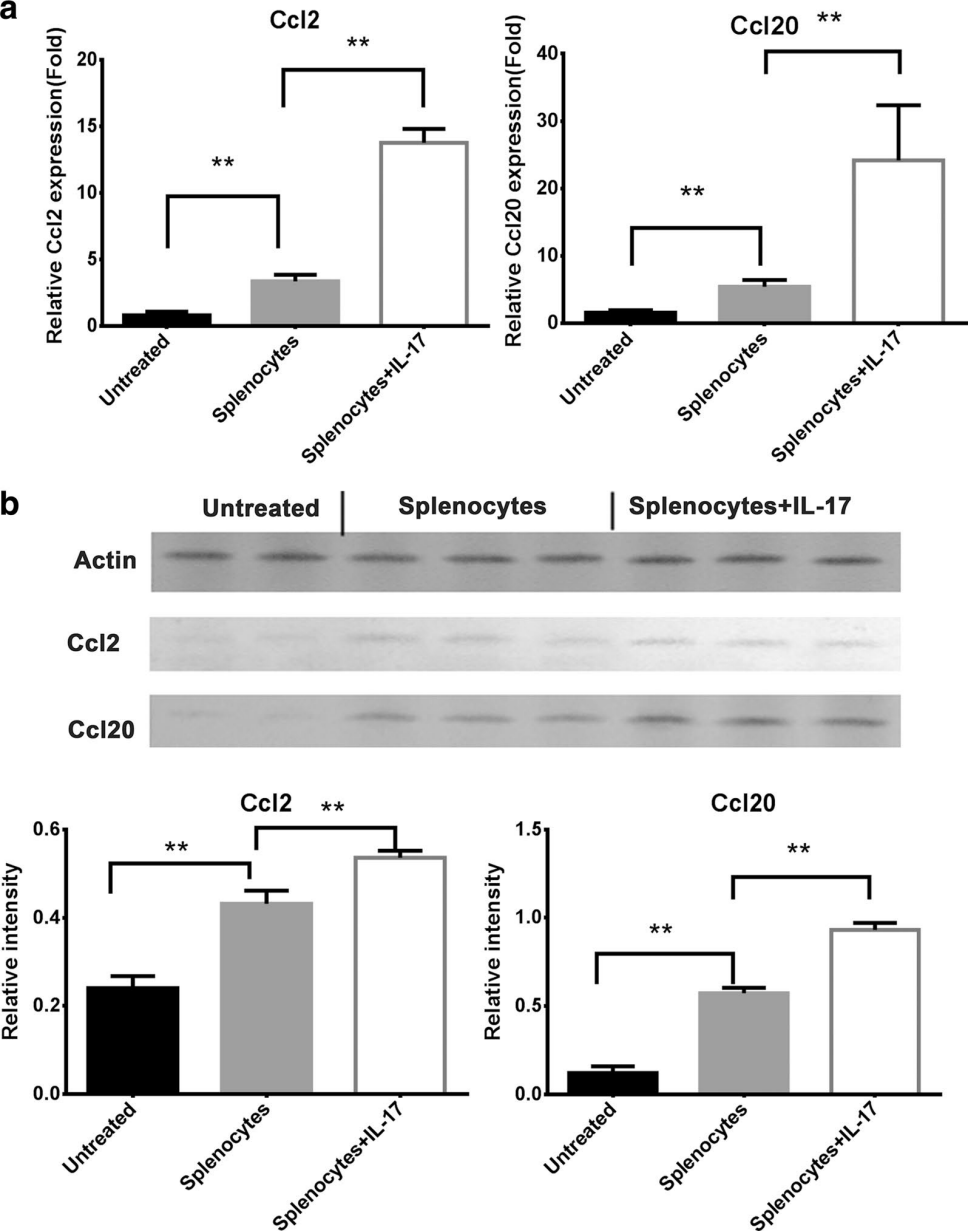
**a** The medulloblastoma xenografts were removed from euthanized mice of all groups on day 28, and cell lysates were isolated for protein and mRNA extraction. Total RNA was extracted, and the mRNA levels of Ccl2 and Ccl20 was analyzed using real-time PCR. Endogenously expressed GAPDH amplification in the same samples was used for a loading control. The experiment was performed three times with consistent results. *p* values were calculated with the *t* test; *p < 0.05; **p < 0.01. **b** Cell lysates were isolated in a protein extraction, and Ccl2 and Ccl20 proteins were analyzed using Western blot. The similar expressions of Ccl2 and Ccl20 proteins in medulloblastoma cells were confirmed by Western blot assay and densitometry assessment. *p* values were calculated with the *t* test; *p < 0.05; **p < 0.01

Because Ccl2 and Ccl20 are critical inflammatory mediators, their expressions were also determined by real-time PCR and Western blot methods on day 28 to investigate additional mechanisms involved in the anti-tumor immunity of IL-17. The results of both real-time PCR and Western blot indicated that the expression levels of Ccl2 and Ccl20 were elevated in the splenocyte-injected group compared to the non-splenocyte-injected group. Moreover, the expression levels of Ccl2 and Ccl20 were higher in the splenocyte-injected group with IL-17 than that without IL-17 (Fig. 4). These results implicated IL-17 may enhance the antitumor effect of splenocyte injection on inflammatory mediators Ccl2 and Ccl20.

### IL-17 was active in IL-17-treated mice

**Fig. 5:**
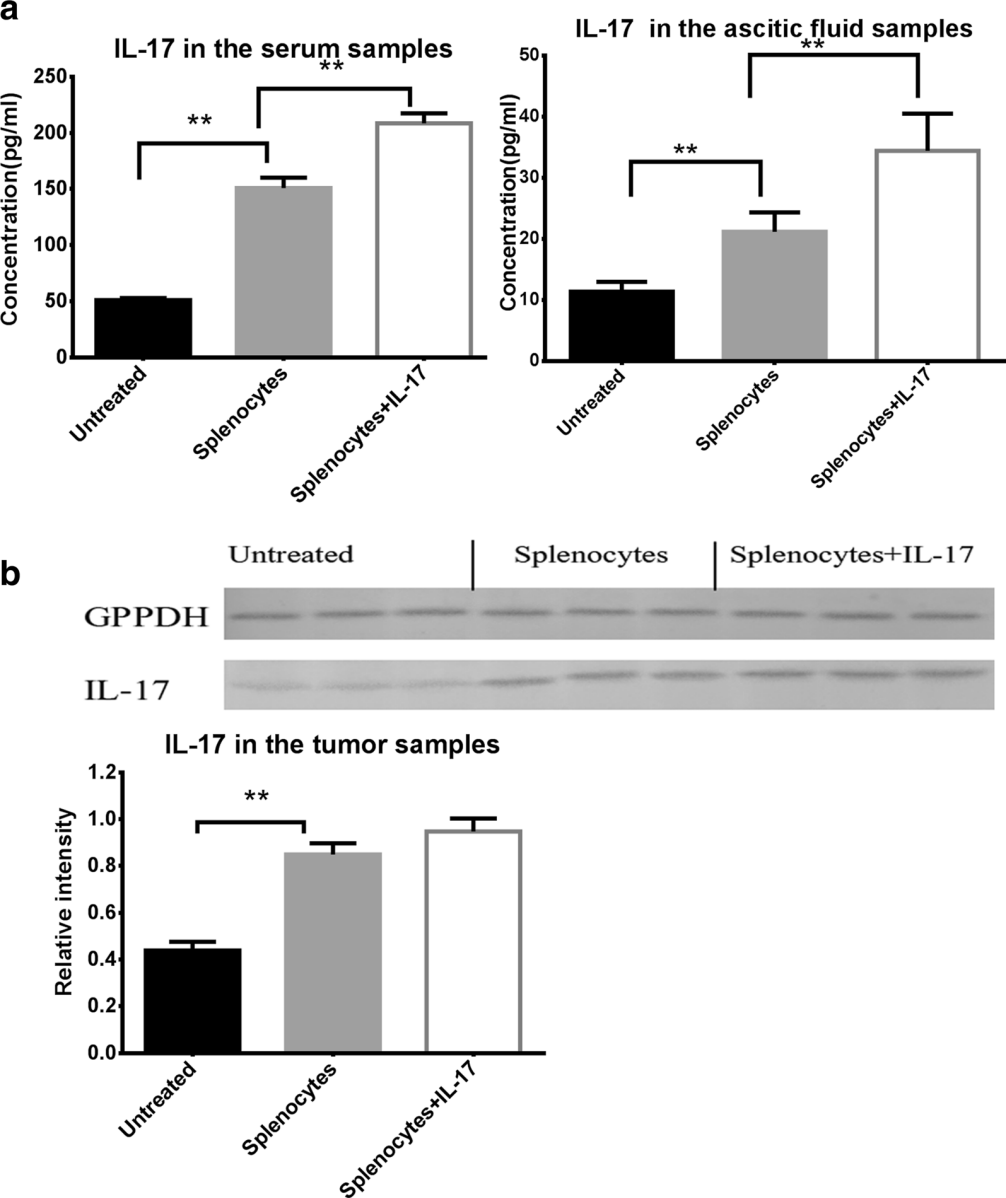
**a** Mean ± SD IL-17 concentrations using enzyme-linked immunosorbent assay demonstrated a statistically significant increase in the levels of IL-17 in serum and ascitic fluid samples in the mice of splenocyte-injected group with IL-17 than that without IL-17. *p* values were calculated with the *t* test; *p < 0.05; **p < 0.01. **b** The medulloblastoma xenografts were removed from mice of all groups on day 14. Cell lysates were isolated in a protein extraction, and IL-17 protein was analyzed using Western blot and densitometry assessment. *p* values were calculated with the *t* test; *p < 0.05; **p < 0.01

We measured the levels of IL-17 in the serum and ascitic fluid samples by ELISA and in the tumor samples by Western blot on day 14 to determine where the cytokine is present and active. As shown in Fig. 5a, the ELISA results obtained from splenocyte-injected group with IL-17 showed that IL-17 levels were higher in the serum and ascitic fluid samples than those without IL-17 (208.54 ± 8.87 vs 152.97 ± 7.01 pg/mL in serum, *P* < 0.01; 34.39 ± 6.09 vs 20.89 ± 3.20 pg/mL in ascitic fluid; *P* < 0.01). Meanwhile, in the non-splenocyte-injected group, the IL-17 levels both in serum (58.38 ± 24.65 pg/mL) and ascitic fluid (12.49 ± 4.02 pg/mL) were the lowest. Whereas, the Western blot results revealed that IL-17 levels were not increased in tumor samples in the IL-17-treated group compared with the non-IL-17-treated group (Fig. 5b). These results implicated that IL-17 may influence tumor-suppressing activity of the splenocytes rather than act directly in the tumors.

### IL-17 enhanced the effect of splenocyte injection on IFN-gamma

**Fig. 6:**
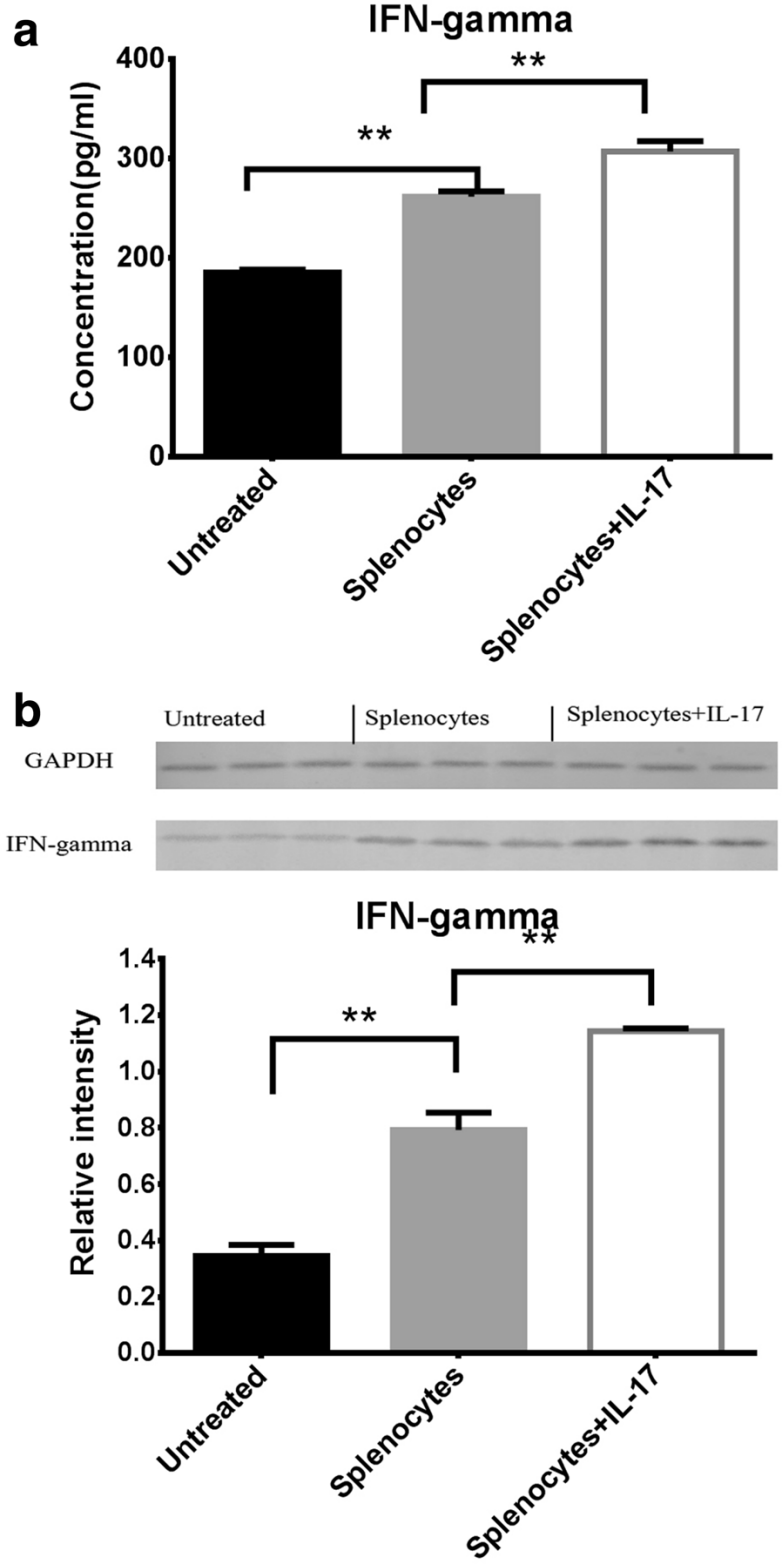
**a** Mean ± SD IFN-gamma concentrations using enzyme-linked immunosorbent assay demonstrated a statistically significant increase in the levels of IFN-gamma in serum samples in the mice of splenocyte-injected group with IL-17 than that without IL-17. *p* values were calculated with the *t* test; *p < 0.05; **p < 0.01. **b** The medulloblastoma xenografts were removed from mice of all groups on day 14. Cell lysates were isolated in a protein extraction, and IFN-gamma protein was analyzed using Western blot and densitometry assessment. *p* values were calculated with the *t* test; *p < 0.05; **p < 0.01

We also assess whether IFN-gamma is expressed by IL-17 stimulated splenocytes that potentially influence the tumor. The levels of IFN-gamma in the serum and tumor samples were determined respectively by ELISA and Western blot on day 14. As shown in Fig. 6a, the ELISA results indicated that the levels of IFN-gamma in serum were elevated in the splenocyte-injected group compared with the non-splenocyte-injected group (261.60 ± 5.73 vs 191.85 ± 22.54 pg/mL, *P* < 0.01), and it was higher in the splenocyte-injected group with IL-17 than that without IL-17 (306.88 ± 10.35 vs 261.60 ± 5.73 pg/mL; *P* < 0.01). The Western blot results showed that the levels of IFN-gamma in tumor samples were in accordance with that in serum (Fig. 6b). These results implicated IL-17 may enhance the anti-tumor effect of splenocyte injection on IFN-gamma.

## Discussion

Medulloblastoma is a high-grade malignant brain tumor with a dismal prognosis, and its current therapeutic treatments are often associated with severe side effects [1–3]. Recent tumor immunology field advances may offer better ways to treat patients with medulloblastoma and reduce the side effects. Th17 cells, found recently, are a new subtype of the CD4+ effector T cell family and characterized as preferential producers of IL-17, IL-21, and IL-22. IL-23 and IL-6 have been shown to play a role in human Th17 cells in vitro [10, 11]. Several studies showed elevated IL-17 and Th17 cells in some types of tumors, such as in ovarian cancer [12, 13]. However, precisely how this might contribute to tumor growth or suppression remains unclear.

Muranski et al. [16] reported that tumor-specific Th17 cells can erase established melanoma in mice. Martin-Orozco et al. [17] also reported that IL-17 and tumor-specific Th17 cells had protective role in antitumor responses by promoting tumor-specific cytotoxic T cell responses. The identification of Th1 cells has facilitated the development of various T cell-based tumor therapies because of its capability of promoting tumor rejection. However, a recent study demonstrated that Th17 may be more effective in promoting tumor rejection than Th1 cells [16]. These findings suggest that Th17 cells may also play an important role in tumor pathogenesis.

Although Th17 cells have been investigated in several types of human tumors, their biological function in tumor development remains elusive. In our previous study, we measured the prevalence of Th17 cells in peripheral blood and tumor-infiltrating lymphocytes and found that increased populations of Th17 cells were present both in medulloblastoma-infiltrating T cells and in patients’ peripheral blood. In addition, the mRNA levels of IL-17, IL-23 and RORC in tumor tissues and the serum concentrations of IL-17 and IL-23 protein were increased in patients with medulloblastoma [18]. Cytokines play an important role in regulating tumor progression and metastasis, and IL-17 is one of the most important Th17 cytokines [10, 11]. Several studies showed that IL-17 has a potent antitumor activity that appears to be T-cell dependent [17, 22]. In contrast, IL-17’s role in promoting tumor growth has also been reported. The tumor-promoting roles of IL-17 may be mediated by angiogenesis [23].

In this study, we used an athymic nude mice model to establish medulloblastoma. We found that, when splenocytes were injected into nude mice, medulloblastoma growth rates decreased compared with the non-splenocyte-injected nude mice. We also found that the medulloblastoma growth rate could be further inhibited in splenocyte-injected mice with IL-17. The results showed that splenocyte injection might inhibit medulloblastoma growth in nude mice. The results also showed that the anti-tumor effects of splenocytes might be enhanced by IL-17.

We also found that IL-17 levels were higher in serum, ascitic fluid samples in the IL-17-treated group than the non-IL-17-treated group. However, IL-17 levels were not increased significantly in tumor tissue samples in the IL-17-treated group. So IL-17 may influence tumor-suppressing activity of the splenocytes rather than act directly in the tumors.

IL-17 is well known for being capable of inducing the secretion of many inflammatory mediators in diverse cell types, including stromal cells and tumor cells [24, 25]. Moreover, cytokines’ roles are highly tumor-type dependent and cell-microenvironment dependent [25]. Th17-related cytokines such as IL-6 and IL-23 are closely related to IL-17. IL-6 and IL-23 have been found in various tumors, including prostate and ovarian tumors as well as colorectal cancers and other malignancies [12–15, 26].

IL-6 was found to increase the proliferation of human tumors transplanted into nude mice and immunocompetent mice [27]. However, IL-6 was also found to inhibit immunogenic tumor growth by stimulating antitumor T-cell activity. IL-6 activates the JAK-STAT3 pathway, leading to increased tumor-infiltrating lymphocytes, which has been widely documented [28]. IL-17 was suggested to activate IL-6 production through activating the AKT signaling pathway. Then, IL-6 promoted signal transducer and activation of transcription 3 (STAT3) signaling and in turn increased IL-8 and VEGF. Supporting these findings, IL-17 promoted STAT3 phosphorylation and lymphocyte infiltration [29, 30].

IL-23 is an important cytokine in Th17 cell regulation. In contrast to our data, IL-23 has been shown to promote tumor progression and prevent anti-tumor immunity [31]. IL-23 produced by macrophages promotes tumor growth by activating STAT-3 in the macrophages and Treg cells [32]. Meanwhile, IL-23 may have a distinct function in cancer immunity by regulating other cells or factors. It is also possible that IL-23 may have different immune functions in different inflammatory settings. IL-23 may provide a supportive role for certain types of tumor chronic inflammation, whereas it might play a role in inhibiting several tumors’ progression in acute inflammation [33, 34].

Thus, in this study, we found that IL-17 up-regulated the expressions of IL-6 and IL-23 in splenocyte-injected nude mice. Moreover, when splenocytes were incubated with IL-17 and injected into athymic nude mice, the medulloblastoma growth rate was decreased compared with the non-IL-17-treated group. So, IL-6 and IL-23 might provide protection to athymic nude mice against medulloblastomas in our current data. The results also suggest that IL-17 in mice might activate splenocytes and/or medulloblastoma cells to produce chemokines Ccl20 and Ccl2, which might promote splenocyte recruitment and anti-tumor activity. IL-17’s effects were thought to be operated through augmented recruitment and anti-tumor activity of splenocytes to medulloblastoma in mice.

IFN-gamma is a cytokine that plays important roles in preventing development of primary and transplanted tumors [35]. Th17 cells can promote the production of IFN-gamma by themselves other Th cells [36]. In this study, the results indicated that the levels of IFN-gamma were elevated in the splenocyte-injected group compared with the non-splenocyte-injected group, and it was higher in the splenocyte-injected group with IL-17 than that without IL-17. Therefore, IFN-gamma might be up-regulated by IL-17 stimulated splenocytes that potentially influence the tumor.

Although splenocyte injection alone did provide some degree of tumor protection in our medulloblastoma model as well, the splenocyte injections with IL-17 were more effective in the medulloblastoma model in athymic mice. IL-17’s effects on tumors are influenced by the immune system, and it inhibits tumor progression in the presence of lymphocytes.

The anti-tumor activities of cytokines and chemokines, like with IL-17, which often depend on the tumor model and local microenvironment, must be taken into consideration when these molecules are used in tumor immunotherapy. In the absence of lymphocytes, though, IL-17 promoted tumor progression. This notion is consistent with our current study on nude mice bearing medulloblastomas with splenocyte injections. The protective function of Th17 cells against tumors is probably due to their ability to enhance inflammatory responses [17].

Punt et al. reported that an increased number of IL-17(+) cells were significantly correlated with the absence of vaso-invasion, smaller tumor size and less infiltration depth in squamous cell carcinoma [37]. Author also reported that a high number of Th17 cells were an independent prognostic factor for improved survival in squamous cervical cancer [38]. Th17 cells are a subpopulation of IL-17(+) cells and had a different correlation with prognosis than total IL-17 [39]. Although Th17 cells had some success in a variety of anti-tumor therapies, in the majority of reported cases it has resulted in limited antitumor responses [16, 17, 40]. We used IL-17 to mediate medulloblastoma rejection. The use of IL-17 is more convenient and secure than using Th17 cells. IL-17 use does not require the culture and identification of specific Th17 cells. Moreover, IL-17 injections may cause fewer allergic reactions compared to T cell injections.

## Conclusions

In conclusion, we found that splenocytes might inhibit the growth rates of medulloblastomas that were transplanted into nude mice, and this inhibition might be enhanced by IL-17. Furthermore, our study showed that injecting IL-17 significantly increased the IFN-gamma, IL-6, IL-23, Ccl2, and Ccl20 chemokine productions in medulloblastoma xenografts in vivo. We speculated that IL-17 might promote IFN-gamma, IL-6, IL-23, CCL-20, and Ccl2 expressions in the subcutaneous medulloblastoma model.

Although our results indicate a probable role for IL-17 in the antitumor activity of splenocytes, we cannot exclude the possibility that other effectors cooperate with splenocytes to inhibit medulloblastoma growth. To illuminate the functions of IL-17 and Th17 on the development of medulloblastoma and other tumors, further research is essential.
